# Prevalent Long-Term Trends of Hypertension in Austria: The Impact of Obesity and Socio-Demography

**DOI:** 10.1371/journal.pone.0140461

**Published:** 2015-10-15

**Authors:** Franziska Großschädl, Erwin Stolz, Hannes Mayerl, Éva Rásky, Wolfgang Freidl, Willibald J. Stronegger

**Affiliations:** 1 Medical University of Graz, Institute of Nursing Science, Billrothgasse 6, 8010, Graz, Austria; 2 Medical University of Graz, Institute of Social Medicine and Epidemiology, Universitätsstraße 6/I, 8010, Graz, Austria; University of Bologna, ITALY

## Abstract

**Background:**

Globally there are only less long-term-studies on hypertension available to provide reliable estimates and identify risk groups. This study aims to analyse the prevalence and long-term-trend of hypertension in Austria, recognize affected subpopulations and investigate social inequalities.

**Methods:**

This representative population-based study is based on self-reported data of adults (mean age: 47.7 ± 17.5; n = 178,818) that were taken from five health surveys between 1973 and 2007. An adjustment of self-reported BMI was performed based on a preliminary validation study. Absolute changes (AC) and aetiologic fractions (AF) were calculated from logistic regressions in order to measure trends. To quantify the extent of social inequality, a relative index of inequality (RII) was computed.

**Results:**

During the study period the age-standardized hypertension prevalence increased from 1.0% to 18.8%, with a considerable rise from 1991 onwards. There was a positive trend in all subpopulations, with the highest AC among obese women (+50.2%) and obese subjects aged 75 years and older (+54.4%), whereas the highest risk was observed among the youngest obese adults (AF: 99.4%). The RII for hypertension was higher for women than men, but in general unstable during the investigation period.

**Conclusions:**

Obesity and older age are significant factors for increased morbidity of hypertension. The most undesirable trends occurred in obese women and obese subjects aged 75 years and older. These risk groups should be given special attention when planning hypertension prevention programs. The high increase in the prevalence of hypertension is due to different aspects, e.g. a demographic change and a change in the definition of hypertension. These findings help to understand why hypertension is becoming more common in the Austrian population.

## Introduction

It is estimated that by the year 2000 about 25% of the adult world population were hypertensive (blood pressure, BP ≥140/90 mmHg) and that this rate will increase to 60% by the year 2025 [1]. For Austria, hypertension has been reported to be a key risk factor for mortality (22.8%), followed by smoking (15.8%), high cholesterol (14.3%), and a high body mass index (BMI) (9.6%) [2].

Excess weight has been found to be associated with an increased prevalence of hypertension, while weight loss dramatically reduces this obesity-related disease [3,4]. The Framingham study estimates that obesity is responsible for 26% of hypertension in men and for 28% in women [5]. The obesity-associated morbidity and mortality of hypertension depends on factors such as sex, age, weight gain and BMI [6,7].

In Austria the age-standardized obesity prevalence strongly increased between 1973 and 2007 (women: 11.5%-15.0%, men: 9.5%-13.8%, p < 0.005) [8]. This may have the potential to impact the risks of cardiovascular diseases. Despite the global increase of obesity [9], only little is known about concurrent trends in the prevalence of cardiovascular risk factors, such as hypertension. Examining socio-demographic subgroups would allow us to identify persons that are particularly at risk [10]. This would facilitate the planning of preventive measures. The aim of our study was to analyse overall long-term trends of hypertension in Austria, among both obese and non-obese subjects, on the basis of selected socio-demographic variables. It was hypothesized that the prevalence of hypertension has risen among Austrian adults in recent decadesand that obese people are particularly affected.

## Method

### Data source and sampling

Health data were deducted from representative national cross-sectional health surveys using similar methodology. They were collected through the Austrian Microcensus in 1973, 1983, 1991 and 1999. The Austrian Health Interview Survey (AT-HIS)–was conducted in 2006–07 instead of the former Microcensus on health as part of the European Health Interview Survey (E-HIS; http://www.euhsid.org). The five surveys were conducted by the federal statistical office `Statistik Austria´ (http://www.statistik.at/web_en/), the owner of the data who makes them available. The Microcensus are chargeable and the data for the AT-HIS are free.

For the Microcensus the sampling was made by a stratified selection of addresses by federal states. The selection framework for the Microcensus sampling was the housing census revised by the current housing statistics in Austria. For the AT-HIS a random sample was drawn from the Austrian population register and stratified by the 32 administrative Austrian districts. In all five surveys data were obtained through standardised face-to-face interviews by trained interviewers of Statistics Austria questioning individuals aged 15 years and older in their private homes or long-term care facilities (such as nursing homes), using interviewer questionnaires. Interviewers of all five surveys had to participate in trainings where they were instructed on how to lead through the interviews. Participants had to give full information for the baseline survey portion. For the Microcensus surveys data from all household members were collected. A sample of the respective individuals was interviewed for the AT-HIS. A further modification in the AT-HIS was, that the participants were questioned by computer-assisted personal face-to-face interviews (CAPI), which allows direct data entry. The raw data were screened for errors from Statistics Austria.

The participation rates were quite high for the Microcensus surveys, especially in 1973, and relatively low in the AT-HIS 2006/07. This is due to the fewer number of questions asked in the first surveys. The questionnaire applied in the AT-HIS was much more extensive in comparison with the questionnaires of the earlier surveys. However, each survey sample was weighted according to sex, age and region to ensure representativeness of the Austrian population distribution.

Data analysis for this study was limited to adults. Subjects aged 20 years and older were included since the AT-HIS survey rather concerned entire age groups in 5 year intervals than exact age levels. Therefore, the data of 64,052 subjects were excluded since they were younger than 20 years at the time of the survey. Furthermore, cases with missing data regarding gender and BMI were excluded (n = 29,709). Cases with implausible BMI values (BMI ≤ 10 kg/m^2^, BMI ≥ 75 kg/m^2^) were also removed from the data base. The proportion of individuals included in the analysis was 63% in total (n = 178,818). 53.7% were female and the mean ± standard deviation (SD) age of the individuals was 47.7 ± 17.5 years old (range: 20–99 years).

### Ethical considerations

This study was approved by the Ethics Committee of the Medical University of Graz (EK-number: 23–172 ex 10/11). It was carried out in compliance with the principles laid down in the Helsinki Declaration. No minors or children were included in the study sample. Data were collected anonymously. Verbal informed consent was obtained from all subjects, witnessed, and formally recorded for every survey.

### Variables and measurement

Self-reported demographic, socioeconomic and health data were collected. In addition, subjects of all surveys were asked whether they had been suffering from hypertension within the last 12 months. In order to measure obesity, participants were asked to indicate their height in centimetres (without shoes) and their weight in kilograms (without clothes). Obesity was defined, according to the World Health Organization (WHO), as a BMI ≥ 30 kg/m^2^. Based on a preliminary validation study [11], BMI correction factors were applied to subjects aged 45 years and older given that the difference between self-reported and measured BMI significantly increased only among older individuals. Correction factors women: 45–59 years = +0.41 kg/m^2^, ≥60 years = +1.09 kg/m^2^. Correction factors men: 45–59 years: +0.50 kg/m^2^, ≥60 years: +0.54 kg/m^2^. Educational level was measured as the highest level reached and categorized as primary school/vocational school (low education); secondary school with general qualification for university entrance (medium-level education); and university/college of higher education (higher education). As the survey of 1973 has failed to collect any educational data, our analysis including educational status refers to the period 1983 to 2007 only.

### Data analysis

Selected variables from the five surveys were fed into a common database. Age-standardized prevalence was calculated based on the WHO’s new European standard population using direct standardization. Binary logistic regression analyses were conducted with hypertension (dichotomous) and the survey period as predictor (correction variable: age in 5-year intervals), across all surveys. To quantify trends in the prevalence of hypertension, percentages of absolute change (AC) were assessed. The aetiologic fraction (AF), a ratio measure, was computed to represent the subgroup with the greatest relative hypertension risk. To calculate the AC and AF, we used the prevalence of the first and last year (Pf and Pl, respectively) as estimated by binary logistic regression models. The AC was defined as Pl-Pf, and the AF as (Pl-Pf)/Pl. The following formulas were applied:

RR=Relative risk(1+exp[−(B0)])/(1+exp[−(B0+B*T)])

AC=1/(1+exp[−(B0+B*T)])−1/(1+exp[–B0])

AF=(RR−1)/RR

B = Regression coefficient; B0 = Intercept; T = Time period in years;

The magnitude of inequalities for hypertension between educational groups was measured by calculating the relative index of inequality (RII). The RII is frequently computed as exponent of the regression coefficient and in this case describes the frequency—or the odds—predicted at the lowest point of the social hierarchy divided by the frequency—or the odds—predicted at the highest point of the social hierarchy [12]. The variable ‘educational level’ was replaced by ‘fractional rank’, which was obtained by ranking the sample according to educational level. The population of each educational level is thus allocated a modified rigid score, which is based on the median of the range in the cumulative spread of the population. By regressing the variable hypertension with the fractional rank (correction variable: age in 5-year-intervals) the exponentiation of the regression coefficient represents the RII (= (exp (B) -1) * 100).

Chi-square tests were computed to analyse statistical significance (two-sided, p<0.05). All analyses were conducted using IBM SPSS^®^ Statistics 22.0 for Windows^®^ (IBM Corp., Armonk, New York) and Stata/SE 11.2 for Windows^®^ (StataCorp., College Station, TX, USA).

## Results

In 2006–2007 the age-standardised prevalence of hypertension was 18.8%. A continuous increase in the prevalence was observed, with the highest rates among obese subjects (2007: 42.6%). Hypertension was slightly higher in women than in men and considerably higher in obese women than in obese men. Viewing the age groups, the prevalence increased with age and subjects aged 75 years and older were most prevalent. This was not true for obese men in the latest survey, which showed the 55–74 year old obese men to be most strongly affected. As for the educational groups, the lowest prevalence was found among highly educated. In women, the presence of hypertension clearly decreased as the educational level increased. Obese women with low education were most strongly affected. For men the stratification by obesity across all educational groups was less pronounced compared to women. A minor difference regarding hypertension prevalence was found for middle- and low-educated men (Table 1).

**Table 1:** Crude and age-standardised prevalence of hypertension in five health surveys among obese and non-obese subjects stratified by sex, age and education.

	P value^a^			1973 (n = 55,841)			1983 (n = 38,835)			1991 (n = 35,093)			1999 (n = 34,731)			2006/07 (n = 14,318)		
				%	%	%	%	%	%	%	%	%	%	%	%	%	%	%
Sex (n)	Total	Obese	Non-obese	Total	Obese	Non-obese	Total	Obese	Non-obese	Total	Obese	Non-obese	Total	Obese	Non-obese	Total	Obese	Non-obese
Age group (n)																		
Educational level (n)																		
**Total crude** (178,818)	p<0.001	p<0.001	p<0.001	1.2	1.9	1.1	1.4	3.9	1.2	2.0	6.4	1.5	6.4	15.8	5.0	20.3	42.6	16.2
**Total age-standardized**	p<0.001	p<0.001	p<0.001	1.0	1.3	1.0	1.4	2.9	1.2	2.0	4.3	1.6	6.1	15.1	5.0	18.8	32.4	16.0
20–34 (50,509)	p<0.001	p<0.001	p<0.001	0.2	0	0.2	0.3	0.1	0.3	0.3	1.0	0.2	0.5	2.1	0.5	3.1	11.5	2.5
35–54 (64,666)	p<0.001	p<0.001	p<0.001	0.7	1.3	0.7	0.8	2.9	0.6	1.4	4.7	1.0	3.7	11.2	2.6	11.7	28.8	9.1
55–74 (49,112)	p<0.001	p<0.001	p<0.001	2.0	2.7	1.9	3.0	4.7	2.7	3.9	8.4	3.0	13.0	21.4	10.7	38.8	57.0	32.8
≥ 75 (14,531)	p<0.001	p<0.001	p<0.001	3.0	3.1	3.0	3.3	7.4	2.9	5.7	9.5	5.3	16.7	23.7	0.9	48.6	61.1	45.8
Low education (100,493)	p<0.001	p<0.001	p<0.001	-^b^	-	-	1.4	2.9	1.2	2.0	4.3	1.6	6.3	11.6	5.1	19.6	32.6	16.5
Medium-range education (14,629)	p<0.001	p<0.001	p<0.001	-	-	-	1.1	3.0	0.9	1.8	2.3	1.7	5.2	11.4	4.6	17.3	33.3	15.1
High education (6,365)	p<0.001	p<0.001	p<0.001	-	-	-	0.4	0.0	0.4	1.5	4.0	1.0	5.3	7.6	4.8	13.7	30.4	12.0
**Women total crude** (96,017)	p<0.001	p<0.001	p<0.001	1.5	2.6	1.4	1.9	5.0	1.5	2.3	7.2	1.8	7.1	18.1	5.4	21.5	46.9	16.6
**Women total age-standardized**	p<0.001	p<0.001	p<0.001	1.1	1.6	1.2	1.7	3.3	1.4	2.1	4.3	1.7	6.3	12.3	5.1	18.7	34.0	15.4
20–34 (25,140)	p<0.001	p<0.001	p<0.001	0.2	0.0	0.3	0.4	0.2	0.4	0.2	0.0	0.2	0.4	2.5	0.3	3.0	12.0	2.3
35–54 (33,168)	p<0.001	p<0.001	p<0.001	0.9	1.8	0.8	1.0	3.6	0.7	1.2	4.3	0.9	3.7	12.1	2.6	11.0	34.1	7.7
55–74 (27,967)	p<0.001	p<0.001	p<0.001	2.6	3.3	2.4	3.6	6.0	3.0	4.8	9.8	3.5	13.7	22.7	10.9	39.0	56.4	32.7
≥ 75 (9,742)	p<0.001	p<0.001	p<0.001	3.6	3.5	3.6	3.9	7.6	3.5	6.0	7.5	5.8	17.9	26.5	16.3	51.4	65.6	47.3
Low education (54,497)	p<0.001	p<0.001	p<0.001	-^b^	-	-	1.7	3.2	1.4	2.2	4.3	1.7	6.6	12.6	5.2	20.2	34.9	16.6
Medium-range education (7,154)	p<0.001	p<0.001	p<0.001	-	-	-	1.2	2.5	0.9	1.4	1.1	1.3	4.9	10.1	4.3	13.5	29.6	11.2
High education (2,779)	p<0.001	p = 0.024	p<0.001	-	-	-	0.1	0.0	0.1	0.9	0.0	0.9	5.0	9.9	4.4	9.4	19.2	8.3
**Men total crude** (82,801)	p<0.001	p<0.001	p<0.001	0.7	0.8	0.7	1.0	2.2	0.9	1.6	5.3	1.3	5.6	12.9	4.6	18.9	37.3	15.8
**Men total age-standardized**	p<0.001	p<0.001	p<0.001	0.7	0.7	0.7	1.0	2.0	0.9	1.7	4.4	1.4	5.7	10.0	4.9	18.6	29.8	16.3
20–34 (25,368)	p<0.001	p<0.001	p<0.001	0.1	0.0	0.1	0.2	1.5	0.2	0.3	1.7	0.3	0.7	1.7	0.6	3.2	11.0	2.6
35–54 (31,498)	p<0.001	p<0.001	p<0.001	0.5	0.7	0.5	0.7	2.2	0.6	1.5	5.1	1.1	3.7	10.5	2.6	12.5	24.2	10.5
55–74 (21,146)	p<0.001	p<0.001	p<0.001	1.3	1.3	1.3	2.2	1.9	2.2	2.8	5.5	2.4	12.1	19.4	10.5	38.6	57.8	32.9
≥ 75 (4,789)	p<0.001	p<0.001	p<0.001	2.0	1.7	2.1	2.0	6.9	1.8	5.1	16.2	4.3	14.0	14.0	14.0	43.2	43.8	43.2
Low education (45,996)	p<0.001	p<0.001	p<0.001	-^b^	-	-	1.1	2.0	1.0	1.6	4.3	1.3	5.8	9.7	4.9	18.5	28.7	16.1
Medium-range education (7,475)	p<0.001	p<0.001	p<0.001	-	-	-	1.0	2.0	0.9	2.2	3.0	2.2	5.6	12.3	5.0	21.4	36.6	19.4
High education (3,586)	p<0.001	p<0.001	p<0.001	-	-	-	0.4	0.0	0.4	1.9	4.2	1.2	5.7	6.7	5.4	16.6	38.9	14.7

^a^according to the chi-square test of period effect

^b^missing data for educational status in 1973

Relative inequalities for hypertension were higher in women than in men and showed a general tendency to increase from 1991 onwards. In total the RIIs were rather unstable (Table 2).

**Table 2:** Relative index of inequality for the prevalence of hypertension between 1983 and 2006/07 per period, by sex, age group, and obesity.

	1983	1991	1999	2006/07
	RII (95% CI)	RII (95% CI)	RII (95% CI)	RII (95% CI)
**Total**	158.0* (28.2, 419.2)	37.6 (-18.5, 132.1)	45.5* (14.4, 85.2)	91.5* (51.2, 142.6)
**Sex** ^**a**^				
Women	253.6* (18.6, 953.6)	379.8* (73.0, 1,230.9)	76.8* (26.9, 146,5)	320.9* (188.2, 514.8)
Men	109.3 (-22.5, 464.9)	-37.5 (-66.9, 18.1)	18.5* (16.7, 68.5)	3.1 (-24.3, 40.4)
**Age group**				
20–49 years	1,318 (-3.7, 20,792.5)	0.6 (-81.3, 441.9)	210.5 (-38.7, 1,373.0)	128.6 (-1.0, 428.2)
50–74 years	88.0 (-50.6, 617.9)	309.3* (22.7, 1265.2)	208.9* (73.6, 449.7)	125.7* (52.0, 235.2)
≥ 75 years	125.7 (-4.4, 432.9)	-2.7 (-47.8, 81.1)	16.6 (-11.1, 15.3)	64.4* (21.9, 129.9)
**Obesity** ^**a**^				
Obese	13.0 (-73.9, 389.3)	-9.0 (-76.0, 244.2)	25.5 (-26.5, 114.5)	-9.6 (-48.6, 58.7)
Non-obese	179.0* (25.0, 522.8)	11.8 (-37.2, 99.1)	21.7 (-7.4, 60.0)	71.4* (31.1, 124.2)

^a^Correction variable for regressions: age interval of 5 years

*statistically significant

**Fig 1:**
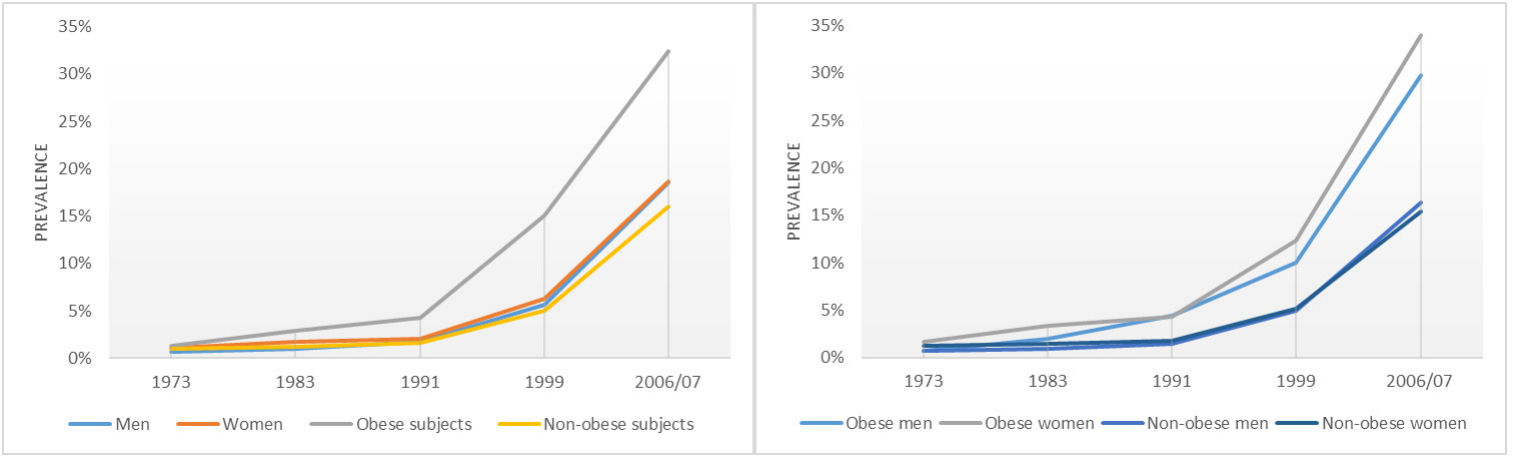
The prevalence of hypertension during the study period, stratified by sex and obesity. Weighted and age-standardised data. All prevalence calculations are statistically significant (p < 0.001).

A strong upward trend in hypertension prevalence was observed, especially from 1991 onwards (Fig 1).

The regression models showed a significant increase in the prevalence of hypertension across all subgroups. For the whole study population, the AC for the prevalence of hypertension was +34.1% (p < 0.001). When distinguishing between obese and non-obese subjects, the highest increase occurred in obese individuals (+37.9%) with maximum scores for women (+50.2%), subjects aged ≥75 years (+54.5%), and those with middle educational level (+24.0%). The highest dynamics for the prevalence of hypertension was measured for obese subjects in the youngest age group (AF = 99.4%) (Table 3).

**Table 3:** Logistic regression of hypertension prevalence change per period as well as absolute changes (AC) and aetiologic fractions (AF) of hypertension during the study period by sex, age, educational status and obesity (adjusted for age). OR = Odds ratio. AC% = absolute change in hypertension prevalence during the study period as computed by logistic regression. AF% = aetiologic fractions of hypertension during the study period as computed by logistic regression.

Predictor	Obese				Non-obese			
	OR hypertension (95% CI)	P-value	AC hypertension (%)	AF hypertension (%)	OR hypertension (95% CI)	P-value	AC hypertension (%)	AF hypertension (%)
**Total**	1.12 (1.12–1.13)	< 0.001	37.9	97.1	1.13 (1.13–1.14)	< 0.001	13.0	96.4
**Sex** ^**a**^								
Women	1.12 (1.11–1.13)	< 0.001	50.2	96.0	1.15 (1.13–1.16)	< 0.001	35.0	94.2
Men	1.15 (1.14–1.16)	< 0.001	18.9	99.0	1.12 (1.12–1.13)	< 0.001	27.3	98.0
**Age group (years)**								
20–34	1.17 (1.11–1.22)	< 0.001	11.5	99.4	1.12 (1.09–1.14)	< 0.001	1.5	93.6
35–54	1.11 (1.10–1.12)	< 0.001	26.6	96.7	1.12 (1.11–1.13)	< 0.001	7.1	96.0
55–74	1.13 (1.12–1.14)	< 0.001	49.7	97.3	1.14 (1.13–1.15)	< 0.001	26.2	96.4
≥ 75	1.13 (1.11–1.15)	< 0.001	54.5	97.0	1.14 (1.12–1.15)	< 0.001	38.3	97.1
**Educational status** ^**a**^ ^**,**^ ^**b**^								
Low education	1.15 (1.14–1.16)	< 0.001	18.0	95.7	1.16 (1.16–1.17)	< 0.001	13.1	96.0
Middle education	1.17 (1.13–1.21)	< 0.001	24.0	96.9	1.18 (1.16–1.21)	< 0.001	14.2	96.7
High education	1.15 (1.09–1.21)	< 0.001	12.2	95.8	1.18 (1.14–1.21)	< 0.001	8.6	97.1

^a^correction variable for regression: age interval of 5 years.

^b^study period = 1983 to 2006/07.

## Discussion

Trends in the prevalence of hypertension reflect the socio-cultural, economic, and demographic impact on the morbidity risk [13]. In our study the prevalence grew steadily, with a strong increase from 1991 onwards. Since the 1990s, BP values have been considered to require treatment from ≥140/90 mmHg onwards. Even earlier, values ≥160/100 mmHg were treated with medication. Thus the number of hypertensive persons suddenly proliferated [14]. In the last years BP target values were revised upward and the trend to low recommendation values for hypertension seems to have been stopped [6,15]. Hypertension guidelines now recommend thresholds of 140/90 mmHg for most people. The values for subjects aged 60 years and older and risk patients were raised (RR ≥150/90 mmHg) [6,15,16]. For mild hypertension (≥140-159/90-99 mmHg), no significant positive effects of medication in primary prevention were found. The damage caused by side effects is likely to be higher than the benefit [17]. The NICE [16] recommend medical therapy for mild hypertension only for subjects with high cardiovascular risk (e.g. end-organ damage). More research is needed regarding the optimal thresholds in hypertension treatment.

Another reason for the strong increase in hypertension prevalence in Austria could be that subjects in the earlier surveys were less often aware of their BP [18], while nowadays many individuals have sphygmomanometers to regularly monitor their BP. Furthermore, the demographic landscape has changed during the study period. There has been a change in the age pyramid in favor of the elderly, which has led to a more frequent occurrence of chronic diseases, including hypertension. The strong rise since the 1990ies in the prevalence of obesity in Austria [8] is most probably associated with the increase in hypertensive inhabitants. In other words, the increasing prevalence of hypertension mirrors the trend of increasing obesity in Austria. When comparing the obesity and hypertension profiles over the study period, the prevalence of hypertension was higher, but a similar increase was found for obesity. A parallel trend and strong relationship between hypertension and obesity was also observed in other studies [19,20].

It was striking that hypertension strongly increased among obese women and obese subjects aged 75 years and older. However, obesity in younger aged men was associated with the highest AFs. This means that the risk for hypertension within the study period was high and that the prevalence threatens to grow even more rapidly in those subgroups.

We found that low-educated, especially obese, subjects were more often hypertensive than subjects with a higher educational status [21]. It was not surprising that the relative inequalities regarding hypertension were higher among women than among men [22]. This may be explained by the fact that among the low education population more women than men suffered from hypertension, whereas the ratio was more balanced in the higher education group. Overall, the RII increased steadily from 1991 onwards. It seems that the increase in hypertension prevalence has led to an increase in relative inequalities. In Austria, only little has been done to reduce such inequalities. Experts recommend the implementation of effective approaches in combination with target-group-specific interventions for hypertension in order to reduce social inequalities [23].

Reportedly greater hypertension prevalence among men than women [20,24] was not found for Austria. In our study the prevalence was higher in women and more pronounced among obese subjects. It should be pointed out that the association between hypertension and obesity is stronger in women than in men [25]. A possible reason is that the visceral fat, that is mainly located in the abdominal area and creates an increased risk for the development of fatal diseases, grows with age. This is more common among women than men [26]. Preventive strategies in Austria should thus be developed from a gender perspective. The increasing prevalence of hypertension among older subjects correlates with a rising BMI at later age in Austria [8]. Therefore, the proportion of elderly obese persons with hypertension is high and was shown to strongly increase during the investigation period. A further reason for the high prevalence of hypertension among older subjects is the fact that the blood vessel elasticity decreases with age. The progressive loss of vascular elasticity especially increases systolic blood pressure. Consequently, elderly patients with progressive vascular stiffness often develop systolic blood pressure values over 140 mmHg, while diastolic values remain under 90 mmHg, i.e. normal. This phenomenon is called increased pulse pressure. The higher the pulse pressure, the more rigid the blood vessels. The majority of hypertensive patients aged 50 years and older have this form of hypertension, which may therefore be justly called a disease of older people that increases with age [4,27]. The observed growing trend for hypertension prevalence among older Austrian adults is also due to the increased proportion of older people during the investigation period, an aspect which has entailed a steady increase ofthe number of chronic diseases including hypertension.

Hypertension is a modifiable and nearly eliminated risk factor that can be counteracted by lifestyle therapy, medication, or both. In Austria most hypertensive patients are treated with medication [28]. A lifestyle modification should be among the first measures taken in the treatment of hypertension, especially when it comes to people with obesity [15]. Weight reduction is the most popular therapeutic intervention, since it soon leads to a lower BP, plasma volume, plasma insulin level, and noradrenalin level. 1 kg less may lead to a reduction of the systolic and diastolic BP of 0.5–2.0 mmHg [29]. Physical activity (at least 30 minutes of moderate dynamic exercise/5-7 days per week) is equally important for a lower BP and reduced left-ventricular hypertrophy. Medical treatment is appropriate for obese patients when relying on antihypertensive medication that blocks or reduces aldosterone action [30]. People tend to overemphasize the achievement of defined BP values and to underestimate the value of preventing hypertensive complications. BP awareness should be created in the population and BP self-measurements should become a preventive routine. Attempts at primary prevention should involve a multifaceted approach based on encouraging the affected groups to adopt a healthy life-style. Antihypertensive medication should support lifestyle changes rather than to replace them [31,32].

According to the Austrian Hypertension League, hypertension is based on at least 30 BP readings. The recommendation “7 out of 30” has led to the outcome: 7 out of 30 = 23.3%: normotension and 8 out of 30 = 26.7%: hypertension [33]. Self-reported hypertension may be biased by a lack of awareness. However, information about the presence was more valid in the case of chronic illnesses and showed adequate validity. Health surveys based on self-reported data have been considered as good instrument for measuring the prevalence of chronic diseases [34]. The disadvantage of self-reported BMI data was compensated by correcting the self-reported BMI [11]. Another limitation was that socioeconomic status was only represented by educational level, as other suitable variables were not available from most surveys. For the calculation of the RII, income would have been a better variable to present the magnitude and to obtain more stable values. The strength of this study comprises the unique database with a very large sample. Thereby it was possible to analyse trends in subgroups and achieve statistically reliable data. The investigation over such a long period allowed an accurate assessment of the development of the hypertension epidemic.

## Conclusion

The prevalence of hypertension strongly increased during the study period. The most undesirable trends occurred in obese women and obese subjects aged 75 years and older. These groups should be given special attention when planning hypertension prevention programs. The presented data suggest that public health strategies implemented to date have fallen short of their targets. This research helps to understand the development of hypertension in Austria and clarifies the need for prevention activities in terms of lifestyle changes in the field of obesity prevention. A multidisciplinary public health approach is necessary, in which gender issues, special risk groups and socioeconomic circumstances should be considered. These strategies should be flanked by a continuous monitoring of hypertension indications.
